# Diaqua­bis­[5-(pyrazin-2-yl)-3-(pyridin-4-yl)-1*H*-1,2,4-triazol-1-ido-κ*N* ^1^]zinc

**DOI:** 10.1107/S1600536812002061

**Published:** 2012-01-25

**Authors:** Bo Li, Peng-Wen Liu, Jing Chen

**Affiliations:** a College of Chemistry, Tianjin Key Laboratory of Structure and Performance for Functional Molecules, Tianjin Normal University, Tianjin 300387, People’s Republic of China

## Abstract

The title mononuclear complex, [Zn(C_11_H_7_N_6_)_2_(H_2_O)_2_], is composed of one Zn^II^ ion, two deprotonated ppt ligands [Hppt = 5-(pyrazin-2-yl)-3-(pyridin-4-yl)-1*H*-1,2,4-triazole] and two coordinating water mol­ecules. The asymmetric unit consists of one half-mol­ecule that is completed by application of a centre of symmetry. The Zn^II^ atom is six-coordinated in an octa­hedral environment, surrounded by two O atoms in the axial positions and four N atoms in the equatorial plane. Adjacent mononuclear units are further linked *via* O—H⋯N hydrogen-bonding inter­actions, forming a two-dimensional network along (100).

## Related literature

For the use of multidentate ligands containing *N*-donor heterocyclic groups in the preparation of metal complexes, see: Du *et al.* (2006 ▶); Li *et al.* (2010 ▶, 2011 ▶); Wang *et al.* (2012 ▶). For crystal structures based on the 5-(pyrazin-2-yl)-3-(pyridin-4-yl)-1*H*-1,2,4-triazole ligand, see: Liu *et al.* (2009 ▶).
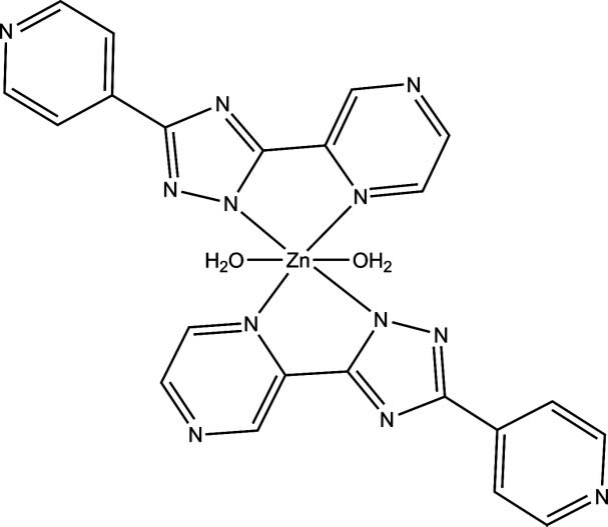


## Experimental

### 

#### Crystal data

[Zn(C_11_H_7_N_6_)_2_(H_2_O)_2_]
*M* *_r_* = 547.85
Monoclinic, 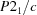

*a* = 10.568 (10) Å
*b* = 12.574 (11) Å
*c* = 9.373 (8) Å
β = 114.483 (14)°
*V* = 1133.5 (17) Å^3^
*Z* = 2
Mo *K*α radiation
μ = 1.13 mm^−1^
*T* = 296 K
0.28 × 0.22 × 0.20 mm

#### Data collection

Bruker SMART CCD area-detector diffractometer
Absorption correction: multi-scan (*SADABS*; Bruker, 1996 ▶) *T*_min_ = 0.742, *T*_max_ = 0.805
5516 measured reflections
1995 independent reflections
1476 reflections with *I* > 2σ(*I*)
*R*_int_ = 0.032

#### Refinement

*R*[*F* ^2^ > 2σ(*F* ^2^)] = 0.035
*wR*(*F* ^2^) = 0.100
*S* = 1.13
1995 reflections
170 parameters
H-atom parameters constrained
Δρ_max_ = 0.78 e Å^−3^
Δρ_min_ = −1.16 e Å^−3^

### 

Data collection: *SMART* (Bruker, 2007 ▶); cell refinement: *SAINT* (Bruker, 2007 ▶); data reduction: *SAINT*; program(s) used to solve structure: *SHELXS97* (Sheldrick, 2008 ▶); program(s) used to refine structure: *SHELXL97* (Sheldrick, 2008 ▶); molecular graphics: *DIAMOND* (Brandenburg, 1999 ▶); software used to prepare material for publication: *SHELXTL* (Sheldrick, 2008 ▶).

## Supplementary Material

Crystal structure: contains datablock(s) I, global. DOI: 10.1107/S1600536812002061/vm2152sup1.cif

Structure factors: contains datablock(s) I. DOI: 10.1107/S1600536812002061/vm2152Isup2.hkl

Additional supplementary materials:  crystallographic information; 3D view; checkCIF report

## Figures and Tables

**Table 1:** Hydrogen-bond geometry (Å, °)

*D*—H⋯*A*	*D*—H	H⋯*A*	*D*⋯*A*	*D*—H⋯*A*
O1—H1*B*⋯N5^i^	0.85	1.99	2.833 (4)	173
O1—H1*A*⋯N2^ii^	0.85	2.13	2.975 (4)	175

Symmetry codes: (i) 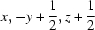; (ii) 
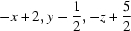
.

**Fig. 1.**
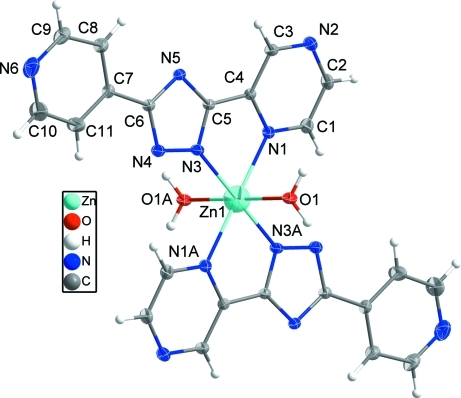
Molecular structure of the title complex showing displacement ellipsoids for all non-H atoms drawn at the 30% probability level. [Symmetry code (A): -x+2, -y, -z+2.]

**Fig. 2.**
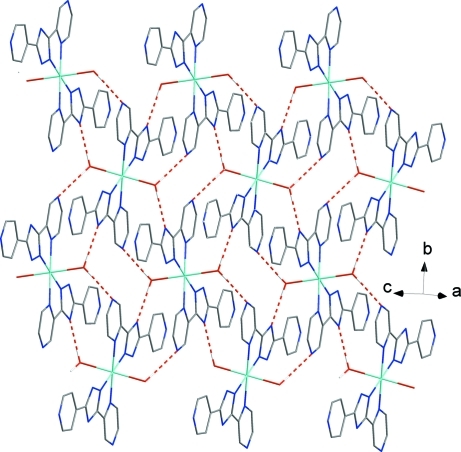
View of the two-dimensional network connected via O–H···N hydrogen bonds (red dashed lines).

## supplementary crystallographic information

### Comment

Multidentate ligands containing *N*-donor heterocyclic groups, such as the pyridyl, pyrazinyl, imidazolyl, oxadiazolyl, and triazolyl (see Du *et al.*, 2006; Li *et al.*, 2010; Li *et al.*, 2011;Wang *et al.*, 2012), have been widely used to prepare diverse metallo supramolecular complexes. In this context, 5-(pyrazin-2-yl)-3-(pyridin-4-yl)-1*H*-1,2,4-triazole (Hppt), an asymmetric ligand with multiple binding sites, has attracted little attention thus far (see Liu *et al.*, 2009). Herein, we report on the title complex [Zn(ppt)_2_(H_2_O)_2_] crystallizing in the monoclinic space group *P*2_1_/*c*, which shows a mononuclear coordination motif and H-bonding supramolecular layers.

The molecular structure of the title complex is centrosymmetric. As illustrated in Fig. 1, the asymmetric unit of this mononuclear complex is provided by a half-occupied Zn^II^ center as well as one deprotonated ppt and one water ligands. The Zn^II^ ion is six-coordinated to four nitrogen atoms from two ppt ligands with the Zn—N distances of 2.040 (3) Å and 2.212 (3) Å, as well as two axial water molecules with the Zn—O distance of 2.252 (3) Å. The deprotonated ppt ligand adopts the chelating coordination mode by using the pyrazinyl and triazolyl groups with the N1—Zn1—N3 angle of 77.52 (11)°.

As shown in Fig. 2, the adjacent mononuclear molecules are further linked to form a two-dimensional network *via* O1—H1A···N2^ii^ [symmetry operation (ii) = -*x+2*, *y*-1/2, -*z*+5/2] hydrogen bonding between the water ligands and the uncoordinated pyrazinyl nitrogen atoms, as well as O1—H1B···N5^i^ [symmetry operation (i) = *x*, -*y*+1/2, *z+*1/2] between the water ligands and 4-position nitrogen atoms of triazole (Table 1). Furthermore, intralayered π···π stacking interactions are also observed between the triazole (N3, N4, N5, C5, C6) and pyrazine (N1, N2, C1—C4) rings with the center-to-center distance of 3.558 (4) Å and dihedral angle between both best planes of 9.5 (2) °, as well as between the triazole ring (N3, N4, N5, C5, C6) and pyridyl groups (N6, C7—C11) with the center-to-center distance of 3.807 (4) Å and dihedral angle of 8.5 (2) °.

### Experimental

A CH_3_OH solution (3 ml) of Hppt (11.2 mg, 0.05 mmol) was carefully layered onto an aqueous solution of Zn(OAc)_2_.2H_2_O (21.9 mg, 0.1 mmol) in a straight glass tube. After evaporating the solvents slowly for *ca* 1 week, colorless block single crystals suitable for X-ray analysis were produced. Analysis, calculated for C_22_H_18_ZnN_12_O_2_: C, 48.23; H, 3.31; N, 30.68%; found: C, 49.05; H, 3.38; N, 30.59%.

### Refinement

All H atoms were initially located in a difference Fourier map, which were then constrained to an ideal geometry, and refined as riding atoms: C—H = 0.93 Å (CH_aromatic_) and O—H = 0.85 Å (OH), with *U*_iso_(H) = 1.2*U*_eq_ (C) and *U*_iso_(H) = 1.5*U*_eq_ (O).

### Crystal data

**Table:**

[Zn(C_11_H_7_N_6_)_2_(H_2_O)_2_]	*F*(000) = 560
*M**_r_* = 547.85	*D*_x_ = 1.605 Mg m^−^^3^
Monoclinic, *P*2_1_/*c*	Mo *K*α radiation, λ = 0.71073 Å
Hall symbol: -P 2ybc	Cell parameters from 2124 reflections
*a* = 10.568 (10) Å	θ = 2.1–27.2°
*b* = 12.574 (11) Å	µ = 1.13 mm^−^^1^
*c* = 9.373 (8) Å	*T* = 296 K
β = 114.483 (14)°	Block, colourless
*V* = 1133.5 (17) Å^3^	0.28 × 0.22 × 0.20 mm
*Z* = 2	

### Data collection

**Table:**

Bruker SMART CCD area-detector diffractometer	1995 independent reflections
Radiation source: fine-focus sealed tube	1476 reflections with *I* > 2σ(*I*)
graphite	*R*_int_ = 0.032
phi and ω scans	θ_max_ = 25.0°, θ_min_ = 2.1°
Absorption correction: multi-scan (*SADABS*; Bruker, 1996)	*h* = −9→12
*T*_min_ = 0.742, *T*_max_ = 0.805	*k* = −12→14
5516 measured reflections	*l* = −11→8

### Refinement

**Table:**

Refinement on *F*^2^	Secondary atom site location: difference Fourier map
Least-squares matrix: full	Hydrogen site location: inferred from neighbouring sites
*R*[*F*^2^ > 2σ(*F*^2^)] = 0.035	H-atom parameters constrained
*wR*(*F*^2^) = 0.100	*w* = 1/[σ^2^(*F*_o_^2^) + (0.0319*P*)^2^ + 1.4706*P*] where *P* = (*F*_o_^2^ + 2*F*_c_^2^)/3
*S* = 1.13	(Δ/σ)_max_ < 0.001
1995 reflections	Δρ_max_ = 0.78 e Å^−^^3^
170 parameters	Δρ_min_ = −1.16 e Å^−^^3^
0 restraints	Extinction correction: *SHELXL97* (Sheldrick, 2008), Fc^*^=kFc[1+0.001xFc^2^λ^3^/sin(2θ)]^-1/4^
Primary atom site location: structure-invariant direct methods	Extinction coefficient: 0.0049 (8)

### Special details

**Table:**

Geometry. All e.s.d.'s (except the e.s.d. in the dihedral angle between two l.s. planes) are estimated using the full covariance matrix. The cell e.s.d.'s are taken into account individually in the estimation of e.s.d.'s in distances, angles and torsion angles; correlations between e.s.d.'s in cell parameters are only used when they are defined by crystal symmetry. An approximate (isotropic) treatment of cell e.s.d.'s is used for estimating e.s.d.'s involving l.s. planes.
Refinement. Refinement of *F*^2^ against ALL reflections. The weighted *R*-factor *wR* and goodness of fit *S* are based on *F*^2^, conventional *R*-factors *R* are based on *F*, with *F* set to zero for negative *F*^2^. The threshold expression of *F*^2^ > σ(*F*^2^) is used only for calculating *R*-factors(gt) *etc*. and is not relevant to the choice of reflections for refinement. *R*-factors based on *F*^2^ are statistically about twice as large as those based on *F*, and *R*- factors based on ALL data will be even larger.

### Fractional atomic coordinates and isotropic or equivalent isotropic displacement parameters (Å^2^)

**Table:**

	*x*	*y*	*z*	*U*_iso_*/*U*_eq_	
Zn1	1.0000	0.0000	1.0000	0.0345 (2)	
O1	0.8507 (3)	0.03619 (18)	1.1088 (3)	0.0401 (7)	
H1A	0.8598	−0.0073	1.1819	0.060*	
H1B	0.8382	0.1011	1.1252	0.060*	
N1	1.0865 (3)	0.1612 (2)	1.0734 (3)	0.0289 (7)	
N2	1.1330 (4)	0.3772 (2)	1.1491 (3)	0.0373 (8)	
N3	0.8842 (3)	0.0902 (2)	0.8093 (3)	0.0315 (7)	
N4	0.7855 (3)	0.0703 (2)	0.6615 (3)	0.0339 (8)	
N5	0.8335 (3)	0.2478 (2)	0.6850 (3)	0.0279 (7)	
N6	0.4516 (4)	0.1950 (3)	0.1161 (4)	0.0556 (10)	
C1	1.1805 (4)	0.1930 (3)	1.2132 (4)	0.0335 (9)	
H1	1.2312	0.1424	1.2872	0.040*	
C2	1.2040 (4)	0.3003 (3)	1.2501 (4)	0.0370 (9)	
H2	1.2712	0.3196	1.3481	0.044*	
C3	1.0395 (4)	0.3452 (3)	1.0079 (4)	0.0346 (10)	
H3	0.9898	0.3963	0.9342	0.042*	
C4	1.0145 (4)	0.2375 (3)	0.9682 (3)	0.0270 (8)	
C5	0.9116 (4)	0.1956 (2)	0.8199 (3)	0.0269 (8)	
C6	0.7575 (4)	0.1661 (2)	0.5912 (4)	0.0286 (8)	
C7	0.6541 (4)	0.1783 (3)	0.4281 (4)	0.0325 (9)	
C8	0.6080 (4)	0.2768 (3)	0.3576 (4)	0.0385 (10)	
H8	0.6421	0.3394	0.4131	0.046*	
C9	0.5105 (5)	0.2796 (3)	0.2035 (4)	0.0462 (11)	
H9	0.4840	0.3461	0.1573	0.055*	
C10	0.4974 (6)	0.1014 (4)	0.1848 (5)	0.0703 (16)	
H10	0.4603	0.0403	0.1264	0.084*	
C11	0.5966 (5)	0.0888 (3)	0.3376 (4)	0.0611 (14)	
H11	0.6241	0.0212	0.3788	0.073*	

### Atomic displacement parameters (Å^2^)

**Table:**

	*U*^11^	*U*^22^	*U*^33^	*U*^12^	*U*^13^	*U*^23^
Zn1	0.0508 (4)	0.0163 (3)	0.0241 (3)	0.0012 (3)	0.0031 (3)	0.0024 (2)
O1	0.064 (2)	0.0205 (12)	0.0351 (13)	0.0068 (12)	0.0202 (13)	0.0035 (10)
N1	0.037 (2)	0.0235 (15)	0.0227 (14)	0.0021 (13)	0.0087 (13)	−0.0003 (11)
N2	0.048 (2)	0.0283 (16)	0.0275 (15)	−0.0072 (14)	0.0081 (14)	−0.0059 (12)
N3	0.044 (2)	0.0180 (15)	0.0244 (14)	−0.0005 (13)	0.0063 (13)	0.0009 (11)
N4	0.046 (2)	0.0230 (15)	0.0229 (14)	−0.0020 (14)	0.0039 (13)	0.0004 (11)
N5	0.0378 (19)	0.0204 (14)	0.0219 (13)	0.0003 (13)	0.0089 (13)	0.0017 (11)
N6	0.058 (3)	0.067 (3)	0.0285 (17)	−0.003 (2)	0.0046 (16)	0.0041 (17)
C1	0.037 (2)	0.033 (2)	0.0236 (17)	0.0025 (17)	0.0055 (16)	0.0005 (14)
C2	0.044 (3)	0.036 (2)	0.0243 (16)	−0.0060 (18)	0.0069 (16)	−0.0065 (15)
C3	0.050 (3)	0.0229 (18)	0.0250 (16)	−0.0016 (16)	0.0094 (18)	0.0020 (14)
C4	0.039 (2)	0.0207 (17)	0.0214 (16)	0.0010 (15)	0.0123 (15)	0.0009 (12)
C5	0.039 (2)	0.0180 (16)	0.0228 (16)	0.0014 (15)	0.0116 (15)	0.0009 (13)
C6	0.037 (2)	0.0223 (17)	0.0244 (16)	−0.0002 (15)	0.0103 (15)	0.0013 (13)
C7	0.042 (2)	0.0292 (19)	0.0235 (16)	−0.0015 (17)	0.0115 (16)	0.0006 (14)
C8	0.047 (3)	0.033 (2)	0.0306 (18)	0.0029 (18)	0.0116 (18)	0.0024 (15)
C9	0.047 (3)	0.052 (3)	0.034 (2)	0.009 (2)	0.0109 (19)	0.0141 (18)
C10	0.090 (4)	0.054 (3)	0.036 (2)	−0.018 (3)	−0.005 (2)	−0.008 (2)
C11	0.088 (4)	0.034 (2)	0.037 (2)	−0.009 (2)	0.001 (2)	0.0009 (18)

### Geometric parameters (Å, °)

**Table:**

Zn1—N3	2.040 (3)	N6—C9	1.329 (5)
Zn1—N3^i^	2.040 (3)	N6—C10	1.332 (6)
Zn1—N1^i^	2.212 (3)	C1—C2	1.390 (5)
Zn1—N1	2.212 (3)	C1—H1	0.9300
Zn1—O1	2.252 (3)	C2—H2	0.9300
Zn1—O1^i^	2.252 (3)	C3—C4	1.401 (5)
O1—H1A	0.8501	C3—H3	0.9300
O1—H1B	0.8502	C4—C5	1.463 (5)
N1—C1	1.336 (4)	C6—C7	1.474 (5)
N1—C4	1.361 (4)	C7—C11	1.388 (5)
N2—C2	1.343 (5)	C7—C8	1.393 (5)
N2—C3	1.343 (4)	C8—C9	1.385 (5)
N3—C5	1.352 (4)	C8—H8	0.9300
N3—N4	1.368 (4)	C9—H9	0.9300
N4—C6	1.346 (4)	C10—C11	1.390 (6)
N5—C5	1.359 (4)	C10—H10	0.9300
N5—C6	1.375 (4)	C11—H11	0.9300
			
N3—Zn1—N3^i^	180.00 (12)	N2—C2—C1	122.2 (3)
N3—Zn1—N1^i^	102.48 (11)	N2—C2—H2	118.9
N3^i^—Zn1—N1^i^	77.52 (11)	C1—C2—H2	118.9
N3—Zn1—N1	77.52 (11)	N2—C3—C4	122.1 (3)
N3^i^—Zn1—N1	102.48 (11)	N2—C3—H3	118.9
N1^i^—Zn1—N1	180.0	C4—C3—H3	118.9
N3—Zn1—O1	90.35 (12)	N1—C4—C3	120.1 (3)
N3^i^—Zn1—O1	89.65 (12)	N1—C4—C5	114.0 (3)
N1^i^—Zn1—O1	92.83 (11)	C3—C4—C5	125.8 (3)
N1—Zn1—O1	87.17 (11)	N3—C5—N5	112.2 (3)
N3—Zn1—O1^i^	89.65 (12)	N3—C5—C4	118.4 (3)
N3^i^—Zn1—O1^i^	90.35 (12)	N5—C5—C4	129.5 (3)
N1^i^—Zn1—O1^i^	87.17 (11)	N4—C6—N5	113.9 (3)
N1—Zn1—O1^i^	92.83 (11)	N4—C6—C7	121.3 (3)
O1—Zn1—O1^i^	180.0	N5—C6—C7	124.8 (3)
Zn1—O1—H1A	111.6	C11—C7—C8	116.9 (3)
Zn1—O1—H1B	117.7	C11—C7—C6	119.9 (3)
H1A—O1—H1B	116.5	C8—C7—C6	123.2 (3)
C1—N1—C4	117.7 (3)	C9—C8—C7	118.7 (3)
C1—N1—Zn1	128.5 (2)	C9—C8—H8	120.7
C4—N1—Zn1	112.7 (2)	C7—C8—H8	120.7
C2—N2—C3	116.6 (3)	N6—C9—C8	125.4 (4)
C5—N3—N4	107.7 (3)	N6—C9—H9	117.3
C5—N3—Zn1	116.6 (2)	C8—C9—H9	117.3
N4—N3—Zn1	135.6 (2)	N6—C10—C11	124.5 (4)
C6—N4—N3	104.6 (3)	N6—C10—H10	117.7
C5—N5—C6	101.6 (3)	C11—C10—H10	117.7
C9—N6—C10	115.2 (3)	C7—C11—C10	119.3 (4)
N1—C1—C2	121.3 (3)	C7—C11—H11	120.3
N1—C1—H1	119.4	C10—C11—H11	120.3
C2—C1—H1	119.4		
			
N3—Zn1—N1—C1	173.8 (3)	N2—C3—C4—C5	177.7 (4)
N3^i^—Zn1—N1—C1	−6.2 (3)	N4—N3—C5—N5	0.8 (4)
O1—Zn1—N1—C1	82.8 (3)	Zn1—N3—C5—N5	177.4 (2)
O1^i^—Zn1—N1—C1	−97.2 (3)	N4—N3—C5—C4	179.8 (3)
N3—Zn1—N1—C4	6.1 (2)	Zn1—N3—C5—C4	−3.6 (4)
N3^i^—Zn1—N1—C4	−173.9 (2)	C6—N5—C5—N3	−0.5 (4)
O1—Zn1—N1—C4	−84.9 (2)	C6—N5—C5—C4	−179.4 (4)
O1^i^—Zn1—N1—C4	95.1 (2)	N1—C4—C5—N3	9.0 (5)
N1^i^—Zn1—N3—C5	178.8 (3)	C3—C4—C5—N3	−169.2 (4)
N1—Zn1—N3—C5	−1.2 (3)	N1—C4—C5—N5	−172.2 (3)
O1—Zn1—N3—C5	85.8 (3)	C3—C4—C5—N5	9.6 (6)
O1^i^—Zn1—N3—C5	−94.2 (3)	N3—N4—C6—N5	0.4 (4)
N1^i^—Zn1—N3—N4	−5.9 (4)	N3—N4—C6—C7	−179.8 (3)
N1—Zn1—N3—N4	174.1 (4)	C5—N5—C6—N4	0.0 (4)
O1—Zn1—N3—N4	−98.8 (4)	C5—N5—C6—C7	−179.8 (3)
O1^i^—Zn1—N3—N4	81.2 (4)	N4—C6—C7—C11	−8.2 (6)
C5—N3—N4—C6	−0.7 (4)	N5—C6—C7—C11	171.6 (4)
Zn1—N3—N4—C6	−176.3 (3)	N4—C6—C7—C8	171.0 (4)
C4—N1—C1—C2	0.1 (5)	N5—C6—C7—C8	−9.2 (6)
Zn1—N1—C1—C2	−167.1 (3)	C11—C7—C8—C9	−0.8 (6)
C3—N2—C2—C1	−1.5 (6)	C6—C7—C8—C9	180.0 (4)
N1—C1—C2—N2	0.8 (6)	C10—N6—C9—C8	−2.9 (7)
C2—N2—C3—C4	1.3 (6)	C7—C8—C9—N6	2.6 (7)
C1—N1—C4—C3	−0.3 (5)	C9—N6—C10—C11	1.5 (8)
Zn1—N1—C4—C3	168.9 (3)	C8—C7—C11—C10	−0.4 (7)
C1—N1—C4—C5	−178.6 (3)	C6—C7—C11—C10	178.9 (5)
Zn1—N1—C4—C5	−9.5 (4)	N6—C10—C11—C7	0.0 (9)
N2—C3—C4—N1	−0.4 (6)		

Symmetry codes: (i) −*x*+2, −*y*, −*z*+2.

### Hydrogen-bond geometry (Å, °)

**Table:**

*D*—H···*A*	*D*—H	H···*A*	*D*···*A*	*D*—H···*A*
O1—H1B···N5^ii^	0.85	1.99	2.833 (4)	173
O1—H1A···N2^iii^	0.85	2.13	2.975 (4)	175

Symmetry codes: (ii) *x*, −*y*+1/2, *z*+1/2; (iii) −*x*+2, *y*−1/2, −*z*+5/2.
